# Serum Osteocalcin and Testosterone Concentrations in Adult Males with or without Primary Osteoporosis: A Meta-Analysis

**DOI:** 10.1155/2017/9892048

**Published:** 2017-08-02

**Authors:** Zhong-Yu Liu, Yang Yang, Chun-Yi Wen, Li-Min Rong

**Affiliations:** ^1^Department of Spine Surgery, The Third Affiliated Hospital of Sun Yat-sen University, Guangzhou, Guangdong 510630, China; ^2^Interdisciplinary Division of Biomedical Engineering, Faculty of Engineering, The Hong Kong Polytechnic University, Kowloon, Hong Kong

## Abstract

Osteocalcin (Ocn) and testosterone play important roles in male skeleton. However, the concentrations of serum osteocalcin and testosterone have never been systematically compared between populations with and without primary male osteoporosis, a common skeletal disorder in adult males. We searched the PubMed, Embase, and Cochrane Library for relevant studies. A meta-analysis was performed to compare the serum osteocalcin and testosterone concentrations between primary osteoporotic males and age-matched nonosteoporotic (non-OP) males. Five case-control studies with 300 adult males were included. We found no significant difference between cases and controls in serum total osteocalcin (TOcn) [95% confidence interval (CI): −1.25, 1.31; *p* = 0.96] and total testosterone (TT) concentrations [95% CI: −0.88, 4.22; *p* = 0.20]. The level of evidence of this carefully performed meta-analysis is 3a according to Oxford (UK) CEBM Levels of Evidence. Future well-designed studies with larger sample size and better standardization of Ocn assay are awaited to confirm and update our current findings.

## 1. Introduction

Osteoporosis (OP) is a systemic skeletal disease characterized by low bone mass and microarchitectural deterioration of bone tissue, with a consequent increase in bone fragility and susceptibility to fracture [1]. Primary osteoporosis consists of two subtypes: type I (postmenopausal osteoporosis) and type II (age-related osteoporosis). Different from the well-studied postmenopausal osteoporosis in females, primary male osteoporosis remains an underdiagnosed and undertreated condition. Osteoporotic fractures result in significant morbidity and mortality in men [2–4].

Mutual dependence exists between bone and gonads [5]. A major risk factor of primary male osteoporosis is the decline of testosterone levels with age in men [6, 7]. Nowadays, the functions of testosterone in bone turnover have been widely studied and interactions are emerging between testosterone and the most abundant noncollagenous protein in bone matrix, osteocalcin. Osteocalcin is mainly synthesized by osteoblasts and is widely used as a marker to indicate the status of bone turnover, although its precise role within the bone matrix remains unclear [8]. Recently, osteocalcin is found to regulate the synthesis of testosterone by Leydig cells in male mice [9], while testosterone is also reported to modulate the osteoblastic expression of osteocalcin in male rats [10].

However, the difference in osteocalcin and testosterone levels between primary osteoporotic males and the age-matched nonosteoporotic (non-OP) males remains unclear, with a few studies providing controversial results [11–16]. Also, few data have been reported to describe the relationship between osteocalcin and testosterone in adult males without metabolic diseases other than primary osteoporosis.

To explore the clinical evidence, we systemically searched and analyzed the available literature to compare the pooled serum osteocalcin and testosterone concentrations between primary osteoporotic males and age-matched non-OP males.

## 2. Materials and Methods

This meta-analysis is strictly subject to the Preferred Reporting Item for Systemic Review and Meta-Analysis (PRISMA) [17, 18] and carried out based on a protocol beforehand, according to the recommendations of the Cochrane Collaboration.

### 2.1. Data Sources and Search Strategies

We searched the online databases of PubMed, Embase, and the Cochrane Library for all the available case-control (osteoporosis versus nonosteoporosis) studies, in December 2015, without restriction to regions, languages, or publication years. The following terms were searched in [title/abstract]:* markers, osteocalcin/bone Gla protein/BGP, male/men, and osteoporosis*. Conventional searches were supplemented by the related articles list. Manual searches of the reference lists of all the relevant studies were also performed accordingly.

### 2.2. Inclusion and Exclusion Criteria

Case-control studies were included if they met the following inclusion criteria: (1) patients in the case group were diagnosed of primary osteoporosis, according to the diagnostic criteria recommended by WHO; (2) people in the control group were age- and sex-matched with those in the case group; (3) osteocalcin as well as testosterone were evaluated in the study. Studies were excluded if they met the following exclusion criteria: (1) the case group consisted of those diagnosed as secondary osteoporosis or under any circumstance that bone turnover, serum osteocalcin, or testosterone level might be affected (e.g., hyperparathyroidism, diabetes mellitus, liver disease, renal insufficiency, antiosteoporotic therapy, and long-term corticosteroid therapy); (2) any group consisted of younger males before or at adolescence (<25 years old); (3) any group consisted of females.

### 2.3. Study Selection and Data Extraction

Two literature reviewers evaluated the eligibility of potential titles and abstracts independently. Included studies were reassessed as full text strictly by inclusion and exclusion criteria. Disagreement was solved by discussion. Further adjudication of a third reviewer was performed if the disagreement remained.

Also independently by two reviewers, using a predesigned form, the following data were extracted: basic information of the study (author's name, nationality, and publication year), demographic information (age, race, and number of people in each group), sampling time, fasting status, and concentration of total serum osteocalcin and testosterone.

### 2.4. Quality Assessment of the Included Studies

Since the design of our included trials was all case-control studies, assessment to the risk of bias was performed using the Newcastle-Ottawa Scale (NOS) [19], recommended by Cochrane Collaboration. A score of 0–9 (allocated as stars) was allocated to each observational study. Studies achieving six or more stars were considered of high quality.

### 2.5. Statistical Analyses

The pooled serum osteocalcin and testosterone concentrations were compared between osteoporotic group and non-OP group. Heterogeneity of the included studies was tested. Heterogeneous data between studies was indicated by *p* ≤ 0.10 or *I*^2^ ≥ 50%, and homogeneous data was indicated by *p* > 0.10 or *I*^2^ < 50%. A fixed effect model was used when the included studies shared a common effect size, whereas a random effects model was used when the effect sizes between studies were not identical. Continuous variables of osteocalcin and testosterone concentrations were reported with mean difference (MD) and 95% confidence interval (95% CI). A *p* < 0.05 was considered to be statistically significant. Statistical analysis was conducted using Review Manager (RevMan 5.3) and SPSS 21. Collected data were carefully inputted and then rechecked by two reviewers respectively.

Subgroup analysis was performed according to different fragments [20–22] and *γ*-carboxylation status [23–25] of the osteocalcin molecules evaluated, if necessary.

## 3. Results

### 3.1. Study Selection

**Figure 1:**
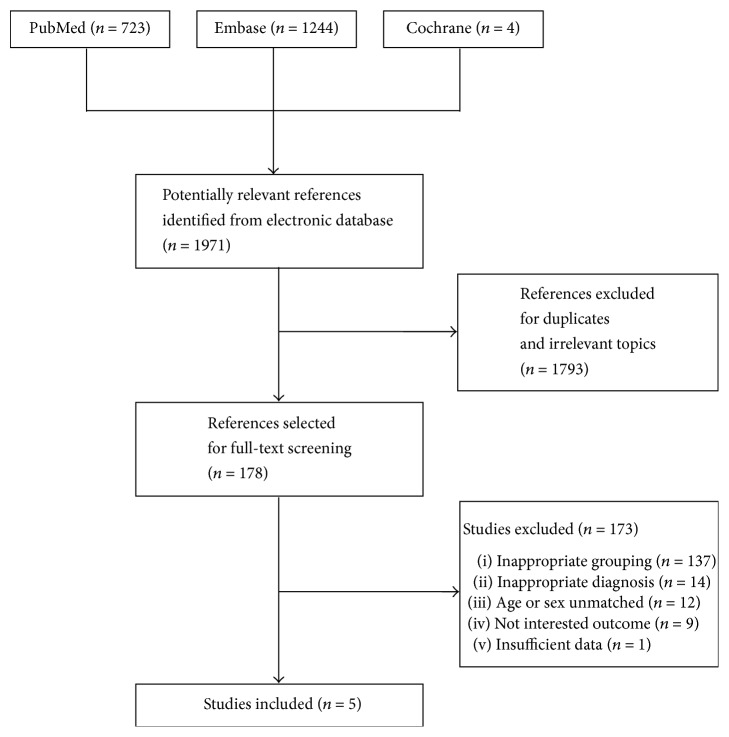
Flow diagram of studies identified, included, and excluded.

1971 references were identified, of which 5 studies [12, 16, 26–28] including 300 adult males (>27 years old) fulfilled all the inclusion criteria and were finally included in this meta-analysis (Figure 1).

### 3.2. Characteristics of Included Studies

Characteristics of the included studies are summarized in Table 1. All the enrolled studies were case-control studies. Serum total osteocalcin (TOcn) and total testosterone (TT) levels were compared, respectively, between osteoporotic group and age-matched non-OP group in all the 5 included studies.

### 3.3. Quality Assessment

Quality assessment of the included studies was summarized in Table 1. Two reviewers independently assessed the included studies and disagreement was solved by discussion. Further adjudication of a third reviewer was performed if the disagreement remained. All the included 5 studies scored ≥7 stars and were considered of high quality.

### 3.4. Outcomes

**Figure 2:**
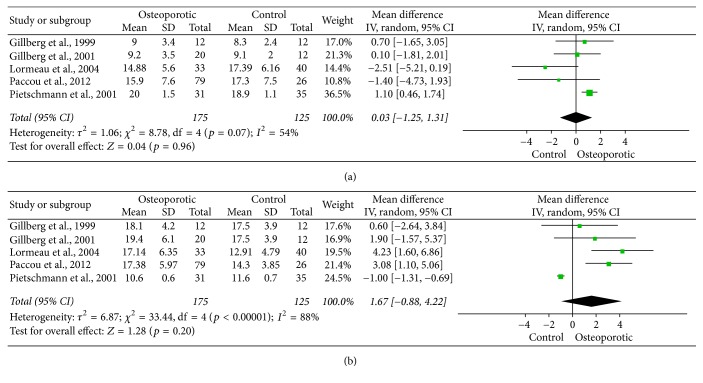
Pooled serum total osteocalcin (a) and total testosterone (b) levels in primary osteoporotic males versus controls.

Significant heterogeneities were found between studies in both TOcn (*p* = 0.07) and TT (*p* < 0.0001) concentrations. As both TOcn and TT levels could be related to age yet the mean ages from different study populations were quite different (Table 1), the effect sizes of TOcn and TT levels in different studies were not identical. Therefore, a random effects model was applied for data synthesis. The mean difference of the pooled TOcn concentrations between osteoporotic and non-OP males was 0.03 *μ*g/L [95% CI: (−1.25, 1.31), *p* = 0.96] and was not statistically significant (Figure 2(a)). Meanwhile, the mean difference of the pooled TT concentrations between osteoporotic and non-OP males was 1.67 nmol/L [95% CI: (−0.88, 4.22), *p* = 0.20], not statistically significant either (Figure 2(b)). However, the data of TOcn was found to present a mirror-inverted distribution with that of TT in the forest plot (Figures 2(a) and 2(b)), indicating potential interactions between the two molecules.

Subgroup analysis according to different fragments and *γ*-carboxylation status of the osteocalcin molecules was not performed, as the osteocalcin assay kits in all the 5 included studies were provided by the same manufacturer (Cis-Bio International, Gif-Sur-Yvette, France) and were all designed to evaluate TOcn.

## 4. Discussion

This meta-analysis was performed carefully and the level of evidence is 3a according to Oxford (UK) Centre for Evidence-based Medicine (CEBM), Levels of Evidence. However, there are several limitations of our study. Firstly, only 5 case-control studies were included with the latest study published about 5 years ago in 2012, due to our strict inclusion criteria and a lack of relevant studies in adult males. The case-control design and inadequate number of recent studies may impact on the level of evidence and reliability of our analysis. Secondly, the individual correlation between the TOcn and TT within all the adult males included was not analyzed, as we could not get the original data from each individual. Instead, a negative ecological correlation between the average TOcn and TT concentrations from each study was observed. However, using aggregate data (ecological correlation) to make inferences about individual subjects (individual correlation) might be fraught with peril (risk of ecological fallacy), and therefore the correlation was not shown in our result. A meta-analysis of the studies directly providing the correlation coefficients of osteocalcin and testosterone could be further carried out for a more convinced result. Moreover, the Ocn molecules in the serum were quite heterogeneous and only uncarboxylated osteocalcin (unOcn) has been found to act as a hormone [29] and interacts with testosterone [9], but all the included studies evaluated serum TOcn (according to the instructions of the assay kits) rather than unOcn. Standardization of Ocn assay and report of the target Ocn molecule is strongly recommended in future clinical studies.

Primary male osteoporosis, which is recognized as a growing public health concern, remains underdiagnosed and undertreated. Different from the high bone turnover status with significantly increased serum osteocalcin level in postmenopausal osteoporosis [30–35] (actually, results from different studies are also controversial and our relevant meta-analysis is still under review in* Biomed Res Int*), bone turnover in primary male osteoporosis is quite complex and heterogeneous [36–38]. Accordingly, we found no significant difference in TOcn levels between primary osteoporotic males and age-matched non-OP controls. This finding suggested that the pattern of bone turnover indicated by TOcn in primary male osteoporosis remains unclear based on the current literature, and the role of TOcn in primary male osteoporosis should be further investigated and validated.

There are 2 types of primary male osteoporosis, age-related osteoporosis, and idiopathic male osteoporosis. Idiopathic male osteoporosis refers to the condition particularly in individuals less than 65–70 years in the absence of an identifiable etiology [39]. Age-related osteoporosis is typically seen in males over the age of 70 years, usually with hormone changes during aging. In the current meta-analysis, no significant difference in TT levels between primary osteoporotic and non-OP males was identified. As a matter of fact, a trend of increase in TT levels in the osteoporotic group was observed in 4 [16, 26–28] of the 5 included studies (Figure 2(b)). This result was quite different from the expectation that the osteoporotic group was more likely to present a lower level of testosterone than the non-OP group. The possible reason was that we had included the individuals with a too wide range of age (>27 years old), while the predominant decline of testosterone was typically seen in age-related osteoporosis in senile males over 70 years old. Therefore, future well-designed studies are needed for a better understanding of the role of testosterone in different types of primary male osteoporosis.

## 5. Conclusions

In summary, this meta-analysis showed no significant difference in TOcn and TT levels between primary osteoporotic males and age-matched non-OP males based on the limited literatures. Much remains unknown in primary male osteoporosis and future well-designed investigations with larger sample size, better standardization of Ocn assay (report of *γ*-carboxylation status and target molecule of Ocn), and further stratification of male OP cases (e.g., according to ages) are needed to confirm and update our current findings.

## Figures and Tables

**Table 1:** Characteristics of included studies.

Study	Sampling condition	Age (year) Mean ± SD (Range)		TOcn (*μ*g/L) Mean ± SD		TT (nmol/L) Mean ± SD		BMI (kg/m^2^) Mean ± SD		Sample size		NOS^a^
		OP	Con.	OP	Con.	OP	Con.	OP	Con.	OP	Con.	
Paccou et al., 2012	8–10 am fasting	54 ± 11 (NA)	50 ± 8 (40–NA)	15.9 ± 7.6	17.3 ± 7.5	17.38 ± 5.97	14.30 ± 3.85	24.4 ± 3.7	27.1 ± 5.0	79	26	★★★★★★★★
Lormeau et al., 2004	7:30–9 am fasting	54 ± 12 (NA)	51 ± 10 (NA)	14.88 ± 5.6	17.39 ± 6.16	17.14 ± 6.35	12.91 ± 4.79	24.51 ± 3.7	26.04 ± 3.6	33	40	★★★★★★★★
Pietschmann et al., 2001	NA	61 ± 11 (40–76)	56 ± 12 (38–76)	20.0 ± 1.5	18.9 ± 1.1	10.6 ± 0.6	11.6 ± 0.7	NA	NA	31	35	★★★★★★★
Gillberg et al., 2001	7-8 am fasting	45 ± 9 (27–57)	43 ± 10 (27–62)	9.2 ± 3.5	9.1 ± 2.0	19.4 ± 6.1	17.5 ± 3.9	23.9 ± 2.6	25.9 ± 3.3	20	12	★★★★★★★
Gillberg et al., 1999	7-8 am fasting	42 ± 9 (27–55)	43 ± 10 (27–62)	9.0 ± 3.4	8.3 ± 2.4	18.1 ± 4.2	17.5 ± 3.9	23.2 ± 2.8	25.9 ± 3.3	12	12	★★★★★★★

OP = osteoporotic group; Con. = control group; TOcn = total osteocalcin; TT = total testosterone; BMI = Body Mass Index; NOS = Newcastle-Ottawa Scale; NA = data not available. ^a^Range: 0–9 stars. Studies achieving six or more stars are considered of high quality.
